# Squamous Epithelial Metaplasia in the Respiratory Tract in Uraemics

**DOI:** 10.1038/bjc.1956.26

**Published:** 1956-06

**Authors:** K. Sanderud

## Abstract

**Images:**


### SQUAMOUS EPITHELIAL METAPLASIA IN THE RESPIRATORY TRACT IN URAEMICS

### A PRELIMINARY REPORT ON A STUDY OF POST MORTEM CASES

K. SANDERUD

*From The Gade Institute, Department of Pathology, University of Bergen, Norway*

Received for publication February 23, 1956

By squamous epithelial metaplasia in the respiratory tract is meant a substitution of columnar cell epithelium by squamous epithelium, stratified in at least 3 distinct cell layers.

Changes of this nature have been observed in connection with both acute and chronic irritations of the respiratory tract (Askanazy, 1919; Niskanen, 1949; Wegelin, 1919; Weller, 1953; Weller, 1955, personal communication), and also in cases of vitamin A deficiency as part of a more general epithelial alteration.

Metaplasia in the respiratory tract is of particular interest as a possible precancerous condition.

In a systematic investigation of epithelium in the respiratory tract in over 200 autopsies, we were struck by unusually marked and widespread metaplastic changes in a young man belonging to an age group where metaplasia was otherwise rare. The patient died of uraemia following a subacute glomerulonephritis. There were no other known causes of epithelial metaplasia which offered a natural explanation of the changes observed in this case.

Our autopsy material was therefore examined with a view to metaplasia in uraemia.

In 45 cases under 20 years of age only six had metaplasia, and one of these six, the above mentioned young man (Case 1), died of uraemia caused by kidney disease.

The adult material is summarized in Table I. In 169 adults there were 44 men and 29 women with uraemia. There appeared to be some preponderance of metaplasia in this group of uraemics compared with its incidence in the total number of cases (Table I). The difference, however, was not significant. Several of these cases had had uraemia of short duration as a terminal stage of some other disease, such as circulatory failure, surgical abdominal diseases, or acute urinary retention.

The material was therefore scrutinized, and those cases of uraemia selected where there was definite clinical and pathological proof as well as laboratory evidence that some serious chronic kidney disease was the cause of the uraemia. Ten men and 7 women fell into this category. Metaplasia was found in the epithelium of the respiratory tract in all of these cases.

The ages of the individuals varied between 22 and 86 years, but several of those concerned were young persons. Different types of chronic kidney disease were the causes of uraemia (Table II).

TABLE I.—*Metaplasia of the Respiratory Tract Epithelium in Uraemic Cases, Compared with the Frequency of Metaplasia in Total Number of* 169 *Post Mortem Cases.*

In cases of chronic kidney disease uraemia, metaplasia was found in 100 per cent. " Grading of metaplasia " refers to the extent of the metaplastic change.

| Grading of metaplasia. | Total material. | | Uraemic cases. | | Chronic kidney disease. | |
|---|---|---|---|---|---|---|
| | M. | F. | M. | F. | M. | F. |
| No metaplasia | 30 | 39 | 12 | 14 | 0 | 0 |
| Metaplasia Grade I | 20 | 17 | 10 | 9 | 4 | 5 |
| Metaplasia Grade II | 17 | 9 | 6 | 3 | 1 | 2 |
| Metaplasia Grade III | 33 | 4 | 16 | 3 | 5 | 0 |
| Total | 100 | 69 | 44 | 29 | 10 | 7 |
| % Metaplasia | 70 | 43·5 | 73 | 51·7 | 100 | 100 |

TABLE II.—*Types of Chronic Kidney Disease Causing Uraemia.*

| Diagnosis. | Number of cases. Male. | Female. |
|---|---|---|
| Chronic cysto-pyelonephritis | 6 | 2 |
| Myelomatosis of the kidneys | 1 | — |
| Hydronephrosis | 1* | 2 |
| Tuberculosis of the kidneys | 1 | 2 |
| Chronic glomerulo-nephritis | 1† | — |
| Polycystic kidneys | — | 1 |
| Total | 10 | 7 |

* Case 2. † Case 3.

It would appear from these findings that kidney disease with uraemia might in one way or another lead to squamous epithelial metaplasia in the respiratory tract.

In some of the patients, particularly the older ones, a number of other causative factors may have been active in the development of metaplasia, e.g. chronic irritation of the mucous membrane of the respiratory tract due to polluted air or chronic and acute disease of the respiratory tract. The case histories of the younger patients, however, do not indicate such additional causes.

In order to elucidate further the possible connection between uraemia and metaplasia of the respiratory tract epithelium, a subsidiary investigation of current autopsies was carried out over a period of two months.

We found 2 more cases where uraemia was the cause of death subsequent to kidney disease. These two were young women (Cases 4 and 5) both with considerable metaplastic changes of the bronchial epithelium (Fig. 3 and 4).

In our studies we have examined several microscopic slides from 11 different locations in the bronchial tree. In the subsequent case reports the metaplasia is graded according to the following criteria :

Grade I : Metaplasia in one location.

Grade II : Metaplasia in two or three locations.

Grade III : Metaplasia in four or more locations.

*Case* 1.

0.93/54. Seventeen-year-old schoolboy.

In October 1953 protein was found in the urine at a routine school examination. Last 2 months—gradual increasing dyspnoea and listlessness, which led to confinement to bed. Died 6 hours after admission to hospital in February 1954.

Autopsy revealed a slim, pale, young man. The kidneys were large (380 and and 360 g.) and showed microscopically the typical picture of a diffuse subacute glomerulonephritis.

Lungs and respiratory tract were almost normal in colour compared with the other very pale organs. In the lower part of the trachea and in the bronchi, small diffuse patches of very pronounced metaplastic changes of the epithelium were found, which had 8–10 distinct flat cell layers (Fig. 1 and 2). Grade III metaplasia.

*Case* 2.

0.470/54. Thirty-one-year-old dairyman.

In 1949 protein in the urine, and later repeated stays in hospital because of uraemia, the last time with haemoptysis, marked hypertension, and signs of myocardial infarction (E.C.G.) Died October 1954. Blood urea 414 mg. per cent. Autopsy revealed pale, oedematous organs with the exception of hyperaemic lungs.

Kidneys : Small granular ; microscopically, pronounced changes showing chronic glomerulonephritis. Infarction in the anterior wall of the heart.

There were extensive metaplastic changes of the epithelial lining of the respiratory tract. Grade III metaplasia.

*Case* 3.

0.487/54. Twenty-two-year-old teacher.

In 1951 protein was traced in the urine during the patient's military service. After a series of stays in hospital, the condition was interpreted as chronic nephritis which lasted until his death in October 1954. Urination, which had always inconvenienced him, was only possible by catheterization during the last days of his life. Blood urea 514 mg. per cent. At autopsy, considerable bilateral hydronephrosis and megalo-ureters were found with almost complete loss of kidney parenchyma. The condition was regarded as a sequel of congenital valve formation in the prostatic section of the urethra. No remains of normal kidney tissue were found microscopically. In the large and small bronchi, the epithelium was to a great extent flattened and stratified, as in Cases 1 and 2. Grade III metaplasia.

*Case* 4.

0.99/55. Eighteen-year-old schoolgirl.

Treated at home for anaemia about 3 months prior to her sudden death February 1955 without any exact diagnosis having been made. Autopsy revealed a very slim young woman. The lungs were relatively hyperaemic compared with the otherwise very pale organs. The kidneys were small, pale and granular with fibrosis and infiltration of lymphocytes and numerous hyalinized glomeruli (chronic glomerulo-nephritis). Blood urea post mortem was 556 mg. per cent

(possibly slightly lower during the last hours of life (Madsen, 1924)). The bronchial epithelium was partly squamous, especially in the upper lobe of the right lung (Fig. 3). Grade II metaplasia.

*Case* 5.

0.108/55. Thirty-five-year-old woman secretary.

In 1944 dysuria and protein in the urine with a progressive insufficiency of kidney function and rising blood pressure until her death in March 1955. Ten days before she died the blood urea level was 582 mg. per cent. Autopsy revealed huge polycystic kidneys (1110 g. and 1600 g.) with no remains of normal parenchyma. There were fibrous tuberculous changes in the periphery of the upper lobe of the right lung only. The bronchi in all sections of the lungs were covered to a considerable extent by a fairly regular 5–7 cell layer thick squamous epithelium (Fig. 4). The subepithelial connective tissue was hyperaemic and oedematous. Grade III metaplasia.

### DISCUSSION

Squamous metaplasia of the bronchial epithelium is not uncommonly seen in routine autopsy material when thoroughly studied, but its presence seems to be rather prevalent in cases of uraemia. In each of 17 cases of chronic kidney disease with uraemia, metaplasia was found to have taken place.

None of these cases suffered from acute respiratory disease which according to the literature, may lead to epithelial metaplasia (Askanazy, 1919 ; Niskanen 1949 ; Wegelin, 1919 ; Weller, 1953.) and which was also present in several other non-uraemic cases with metaplasia.

As already mentioned, we cannot exclude the possibility that chronic irritants of various types (such as tobacco smoke and pollution of air by dust) to some extent may have affected these cases, particularly amongst the older patients. Neither can we, in spite of the negative information, completely ignore the possible influence of some chronic or acute respiratory infection.

It would appear that the role of irritants is of minor importance in the younger age group, and in none of 5 cases reported in detail did the previous history suggest any kind of irritation of the respiratory tract epithelium. In these cases, therefore, we must look for other explanations of the epithelial changes.

It is well known that considerable general, squamous epithelial metaplasia may develop in many organs, also extensively in the respiratory tract in cases of vitamin A deficiency (Sweet, 1935 ; Wolbach and Howe, 1925). It is possible to presume that kidney disease interferes in a decisive manner with the metabolism of vitamin A and thereby leads to metaplasia of the epithelium. The vitamin may be destroyed, or the absorption, transportation or storage interfered with, or the synthesis may be blocked in some way or other. Our investigations did not, however, elucidate these problems.

The uraemic condition in kidney failure, on the other hand, often leads to considerable pathological changes which may not have anything to do with the metabolism of vitamin A. Both uraemic pericarditis and uraemic colitis are well known conditions, the latter possibly due to vascular changes, which can often be demonstrated in the uraemic cases (Siegmund, Henke and Lubarsch, 1929), and which may lead to ulceration and necrosis of the intestinal mucosa. " The uraemic lung " is also a well known term, and changes may be found both by X-ray examination and morphologically in this condition (Bass and Singer, 1950; Ehrich and McIntosh, 1932). Microscopic examination of such lungs, reveals thickening of the alveolar septa, hyperaemia and fibrinous exudate. Bronchiolitis obliterans has been regarded as a possible last stage of such processes (Bass and Singer, 1950).

One reason why epithelial metaplasia has not been observed previously in these lungs may possibly be that autolysis of the bronchial epithelium takes place very rapidly if the material is not fixed at an early stage post-mortem. It is possible that the alteration of circulation, which to a great extent takes place in some uraemic cases, may lead to trophic disturbances of the mucosa with metaplasia as a result. The manner of differentiation of the respiratory tract epithelium may change, or perhaps ulceration occur, similar to that in the colon in uraemic colitis. Such ulcers will naturally heal with squamous epithelium which may be stratified in several layers and also irregular.

A direct influence on the epithelium from metabolic products in chronic kidney disease may also be taken into consideration. In this connection it may be mentioned that sarcomatous and adenomatous lung tumours have been produced by urethane injections in mice and rats. Urethane is chemically closely related to urea, which may be seen from the formulae :

Urea.

Urethane.

We cannot give any definite explanation of the cause of the epithelial metaplasia in our cases. The mechanism may equally well be interference with the vitamin A metabolism, chronic irritation and possible ulceration due to circulatory disturbance, or an " intrinsic " factor of metabolic origin due to the chronic kidney insufficiency. A combination of these factors may also come into consideration.

### SUMMARY

1. In a comprehensive study of autopsy material, squamous epithelial metaplasia in the respiratory tract was observed remarkably often in individuals with uraemia, particularly in cases where the uraemia was a result of chronic or subacute kidney disease.

2. In the older patients some kind of chronic irritation, not related to the uraemia, may have played a role as the cause of the metaplasia, but in 5 young

---

### EXPLANATION OF PLATES

FIG. 1.—Case 1. Squamous epithelium of lower trachea. H. & E. × 115.

FIG. 2.—Same case. H. & E. × 700.

FIG. 3.—Case 4. Squamous epithelium of upper right lobe bronchus. Duct of submucous gland is blocked by epithelium. H. & E. × 210.

FIG. 4.—Case 5. Squamous epithelium of upper left lobe bronchus. H. & E. × 420.

1

2

Sanderud.

3

4

patients, chronic irritation could be ruled out. In these 5 cases with chronic disease of the kidneys, widespread metaplastic changes were found in the bronchi.

3. Possible causes of the epithelial changes in this condition are discussed.

### REFERENCES

ASKANAZY, M.—(1919) *KorrespBl. schweiz. Ärz.*, **44**, 465.
BASS, H. E. AND SINGER, E.—(1950) *J. Amer. med. Ass.*, **144**, 819.
EHRICH, W. AND MCINTOSH, J. F.—(1932) *Arch. Path.*, **13**, 69.
MADSEN, St. Tschudi—(1924) Studier over restkvelstoffets og urinstoffets optraeden og fordeling specielt i den syke organisme. Bergen (J. W. Eide).
NISKANEN, K. O.—(1949) *Act. Path. Scand.*, Suppl. 80.
SEIGMUND, H., HENKE, F. AND LUBARSCH, O.—(1929) 'Handbuch der speziellen pathologischen Anatomie und Histologie,' IV/3 : 345, Berlin. (Julius Springer.)
SWEET, L. K. AND K'ANG, H. S.—(1935) *Amer. J. Dis. Child.*, **50**, 699.
WEGELIN, C.—(1919) *KorrespBl. schweiz. Ärz.*, **44**, 65.
WELLER, R. W.—(1953) *Amer. J. clin. Path.*, **23**, 768.
WOLBACH, S. B. AND HOWE, P. R.—(1925) *J. exp. Med.*, **42**, 753.


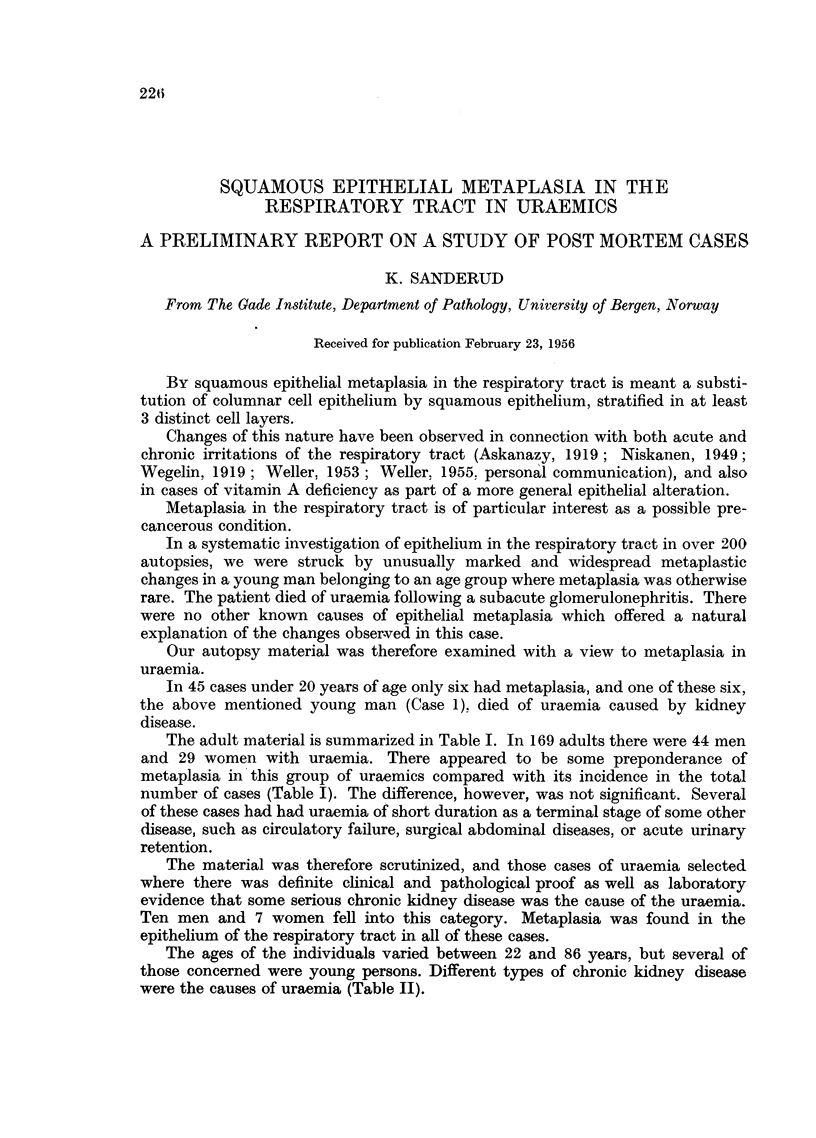

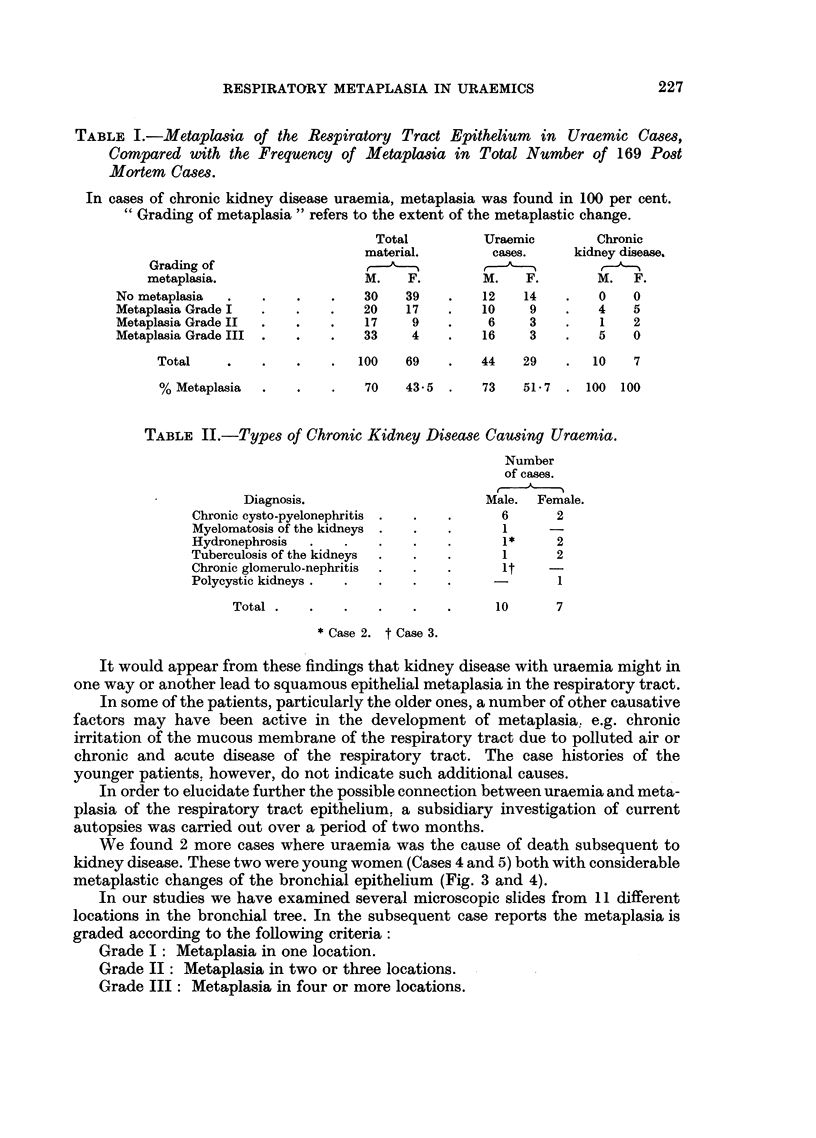

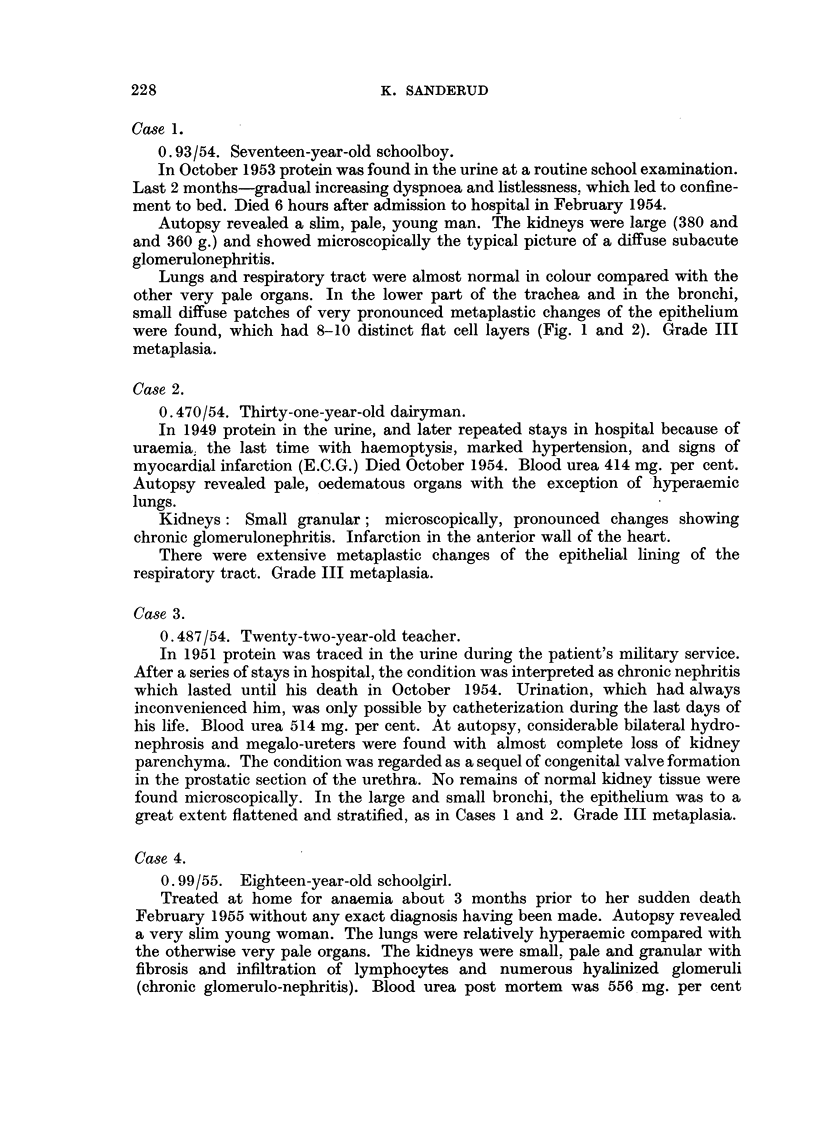

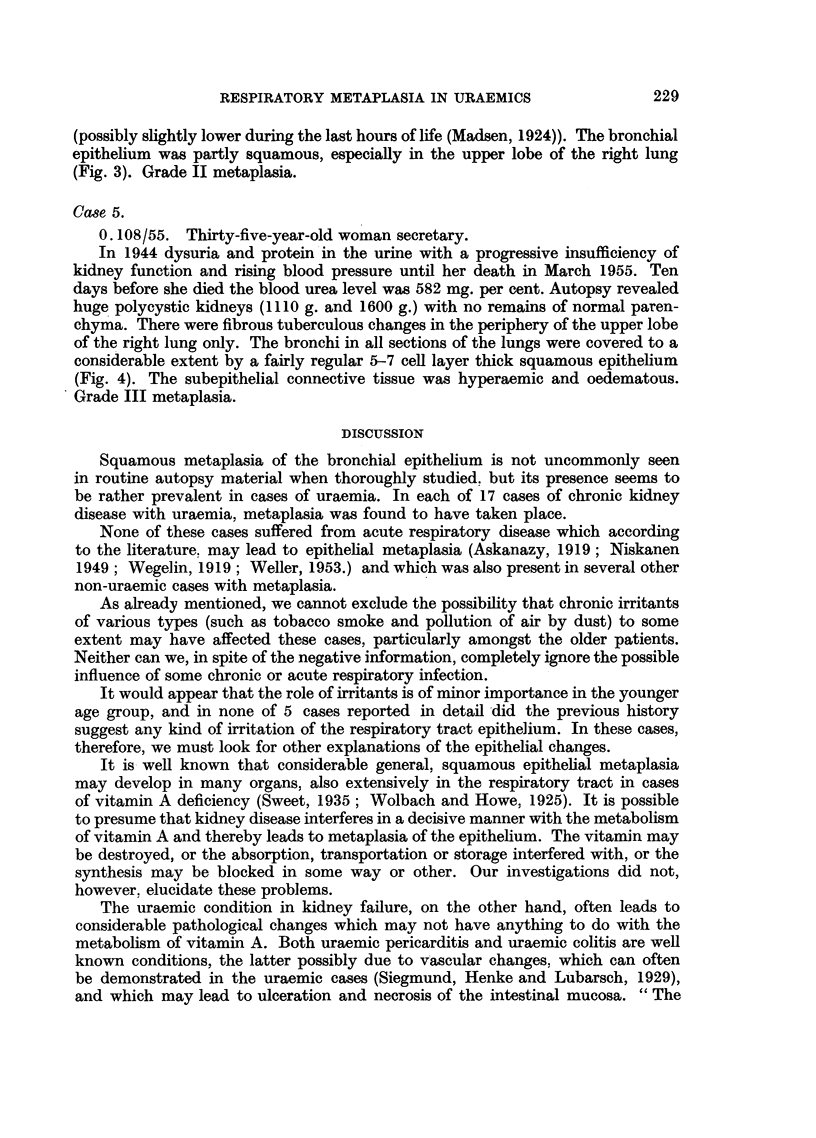

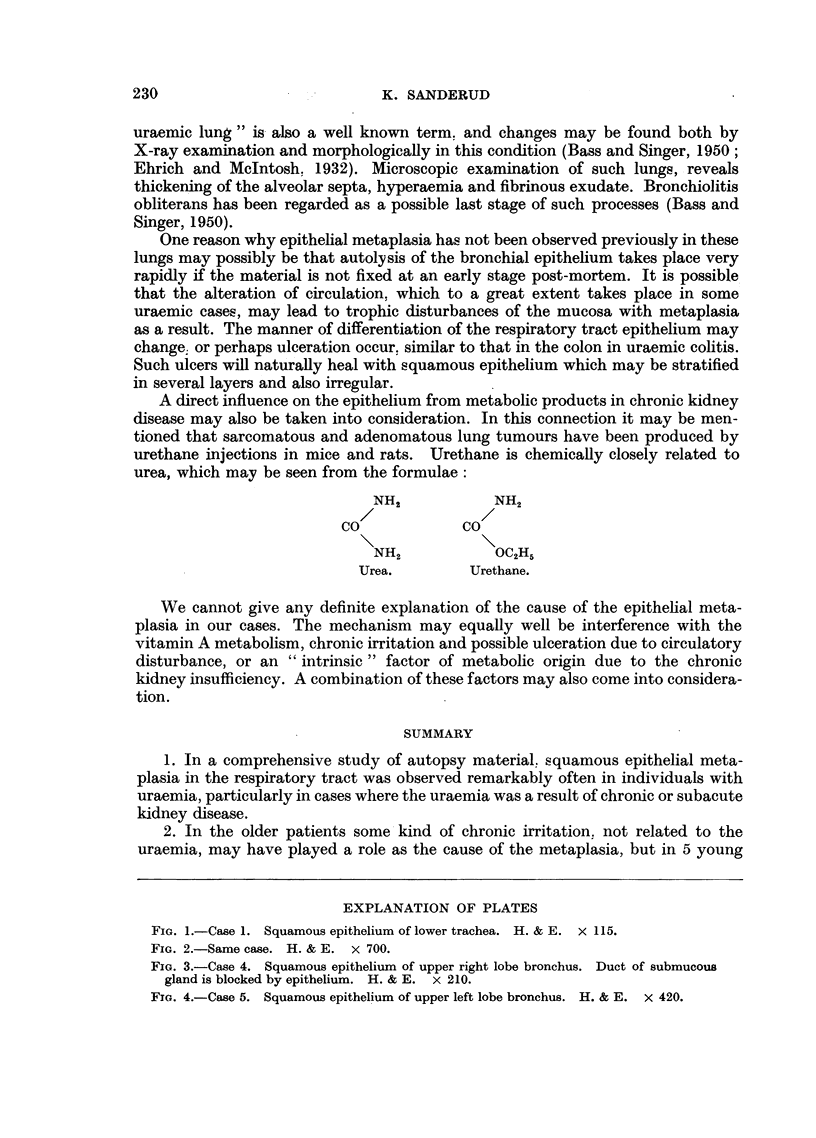

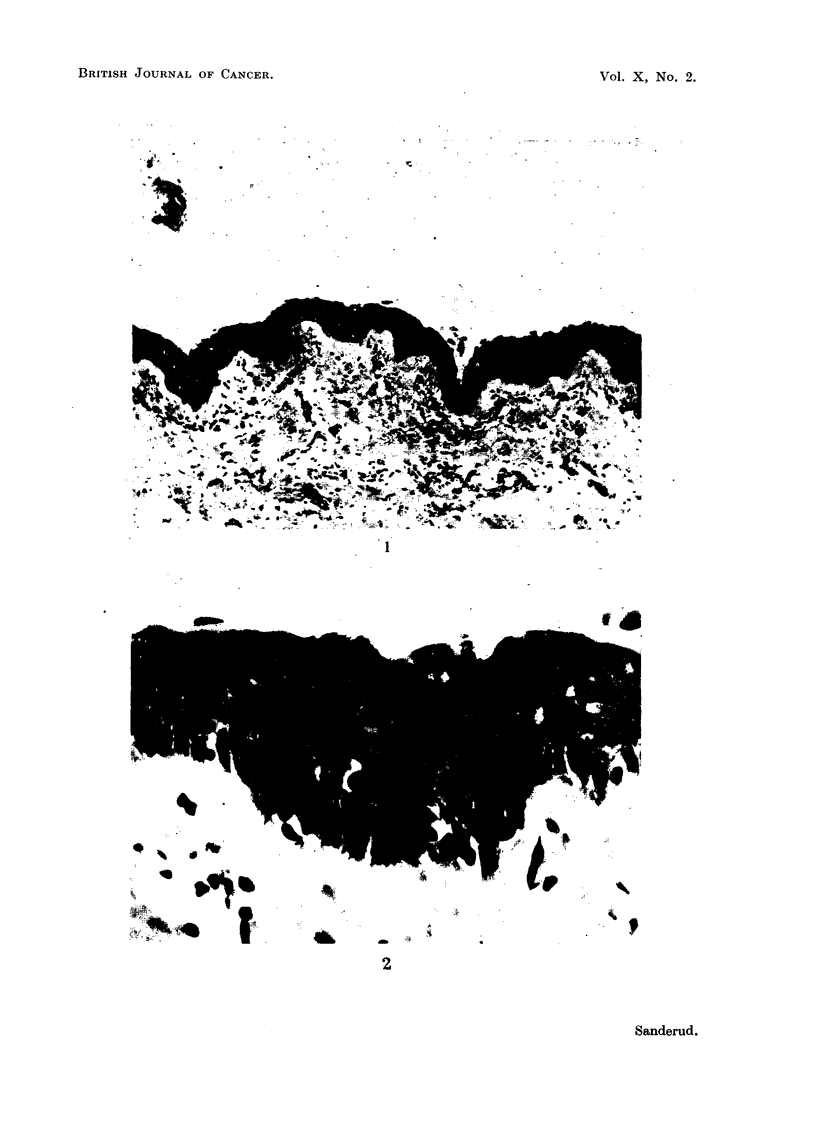

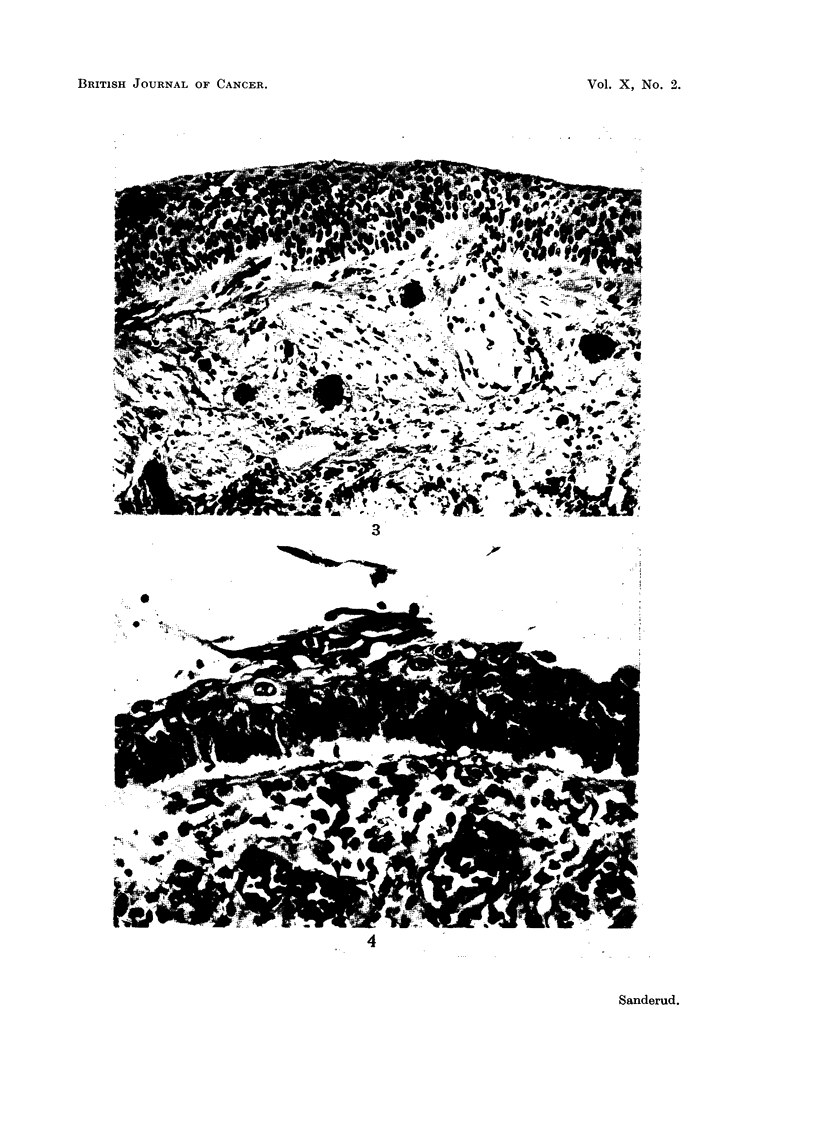

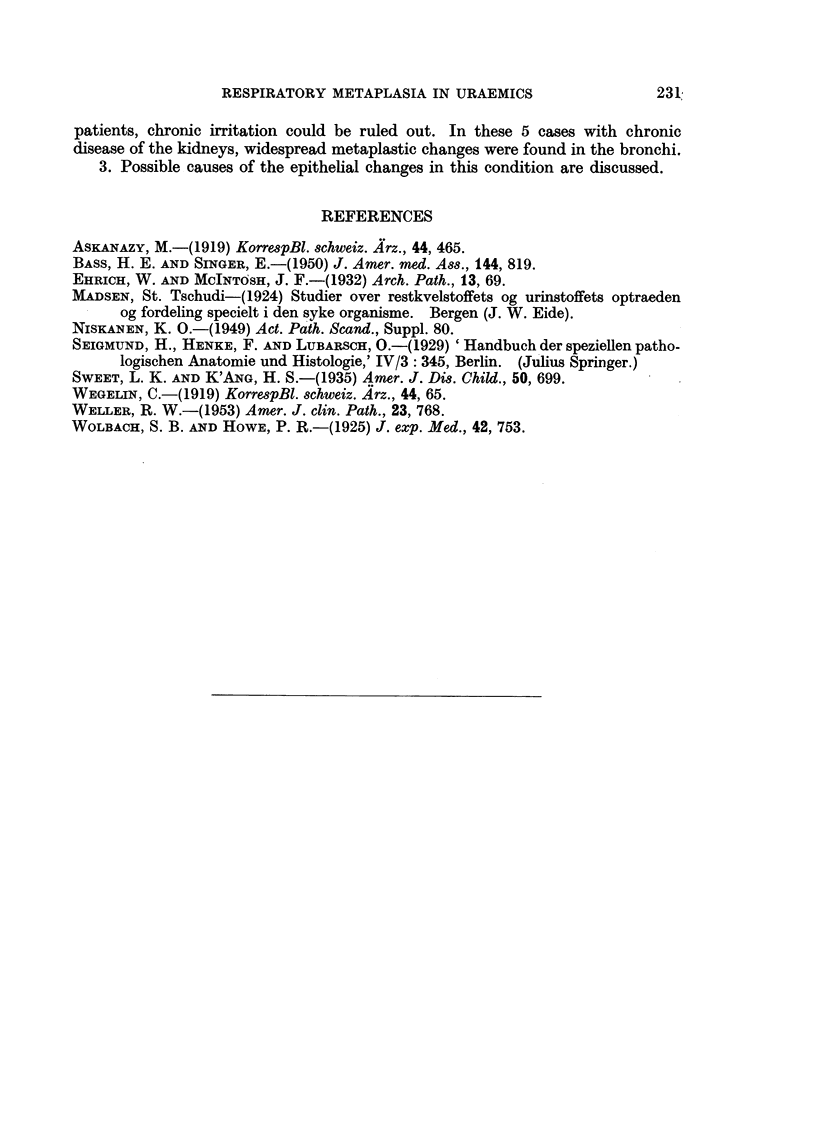


## References

[OCR_00358] BASS H. E., SINGER E. (1950). Pulmonary changes in uremia.. J Am Med Assoc.

[OCR_00370] WELLER R. W. (1953). Metaplasia of bronchial epithelium; a postmortem study.. Am J Clin Pathol.

